# Bashing irreproducibility with shournal

**DOI:** 10.1038/s41598-024-53811-9

**Published:** 2024-02-28

**Authors:** Tycho Kirchner, Konstantin Riege, Steve Hoffmann

**Affiliations:** Computational Biology Group, Leibniz Institute on Aging — Fritz Lipmann Institute (FLI), 07745 Jena, Germany

**Keywords:** Computational platforms and environments, Data publication and archiving, Computational science, Software

## Abstract

Arguably, the most important tool for many computational scientists is the Linux shell. Processing steps carried out there are critical for a large number of analyses. While the manual documentation of the work is time-consuming and error-prone, existing tools do not integrate well into the shell or suffer from a large overhead. Here, we present *shournal*, which integrates tightly into the shell and automatically records all shell commands along with their associated file events. Thus, for all files, it can later be told how they were generated and processed. Additionally, it allows the creation of detailed reports for whole project folders. *shournal* retrieves its data directly from the Linux kernel and allows the monitoring of whole process trees with low overhead.

## Introduction

The daily work of many computational biologists, physicists, meteorologists, data scientists, and many others involves the Linux shell. Despite its limited graphic capabilities and complex syntax, its unmatched flexibility makes it the tool of choice for many file operations, such as sorting or concatenation, as well as for writing small scripts and pipelines. A growing set of software is solely controllable via the command line, which is especially true for the most recent advancements in computational science. However, in larger analysis projects, it quickly becomes challenging to keep track of the work process, as a typical shell workflow involves executing commands with many parameters, modifying scripts, and editing configuration files, usually in an iterative manner. Manually documenting all steps is an effort that is generally only spent for results the researcher considers valuable at execution time or shortly afterward. Otherwise, the shell’s history is often the only way to manually reconstruct the chain of commands that led to a given outcome. This process is rather time-consuming, error-prone, and leaves some critical blind spots, especially in cases where many similar commands were entered. A reconstruction can quickly become impossible if the used scripts or configuration files are not available anymore or have been changed without proper version control. For smaller ad-hoc shell, awk, Perl, or Python scripts, such version control is often omitted, inherently posing a threat to the reproducibility of the work.

In recent years, a plethora of tools have been developed to address the general problem of computational reproducibility^1,2^, a critical subset being the reproduction of pipelines across platforms to share them with other researchers. Prominent tools include workflow engines like *Snakemake*^3^ or *Nextflow*^4^, which provide their own language to describe pipelines and setup routines. For practical or motivational reasons, however, analysis projects are not always directly initiated or consequently carried out within these frameworks. Clearly, the additional work required for using these powerful tools may be deemed excessive for smaller ad-hoc projects or certain pre-processing steps.

Tools like *CDE*^5^, *CARE*^6^, or *ReproZip*^7^ do not require a substantial modification of the user’s workflow. Instead, based on system tracing using the Linux built-in *ptrace*, they can automatically create an archive of the whole pipeline and its dependencies. This archive is executable on another unconfigured computer, alleviating the process of sharing a result with the scientific community. However, due to the high runtime- and storage overhead (see our benchmark below), continuous usage for all shell commands is not advisable, at least not for computationally demanding pipelines. Thus, we assume that in practice, such archives are only created at important milestones of a research project, requiring all intermediate steps to be reproducible on the local machine. As outlined above, achieving local reproducibility can be quite challenging, especially, when several days or weeks have passed until those archivers run, discouraging researchers from creating reproducible pipelines.

Another class of tools are so-called *record-and-replay systems* like *Arnold*^8^ or *rr* (Record–Replay)^9^, which aim at recording entire process trees with plenty of details, e.g., used system calls and their return value, to allow “replaying” them at a later time. However, while being very powerful and suitable for, e.g., debugging, forensics, or finding data races, the amount of necessary tracing and logging to replay processes may be too high when computationally demanding pipelines are monitored on a regular basis. For instance, *Arnold* reports a runtime overhead of up to 100% for a simple CVS checkout and 1 TB of disk space per year and workstation. Further, in order to use *Arnold*, a custom Linux kernel must be compiled, constituting a high burden for administrators or users. The more recent *rr* is based on an optimized *ptrace* using *seccomp-bpf* filters and does not require a custom kernel. However, it reports an almost eightfold runtime overhead for the example of compiling source code using *make*.

SPADE^10^ allows recording provenance on the system-level using the Linux kernel’s builtin *auditd* infrastructure. While it does not record shell commands, such functionality could be added by a plugin. However, while the authors report a runtime overhead of about 10% when monitoring a web-server or a genomic sequence alignment, we find overheads partially exceeding 100% in our own benchmark, discouraging us from using SPADE as tracing backend.

The bash plugin of *Burrito*^11^ associates shell commands and their used files based on tracing with *SystemTap*^12^. However, it yields a runtime overhead of partially more than 20% in our benchmark, which is, as we demonstrate later, substantially higher than necessary. Further, *Burrito* relies on the NILFS versioning filesystem^13^ to save previous file versions, posing a severe limitation for institutions built around network filesystems such as NFS . Finally, *Burrito*’s unconditional preservation of all file versions could quickly result in an unacceptable storage overhead.

Here, we present *shournal*, a configurable and integrated tracker for the bourne again shell (bash) and z shell (zsh).

## Results

*shournal*, the *shell journal*, keeps track of the provenance of files^14^ by continuously recording all shell commands reading or writing them. The association is based on tracing within (Linux-)kernel space. *shournal* is designed from scratch for low overheads to allow being enabled unconditionally for all the user’s shell sessions, even when tracing computationally highly demanding pipelines using terabyte-sized files. According to our benchmark, *shournal* induces a runtime overhead of less than 0.5% while the storage overhead is small, i.e., a few megabytes in case of tens of thousands of file events. Overheads are kept low using three key concepts

**Figure 1:**
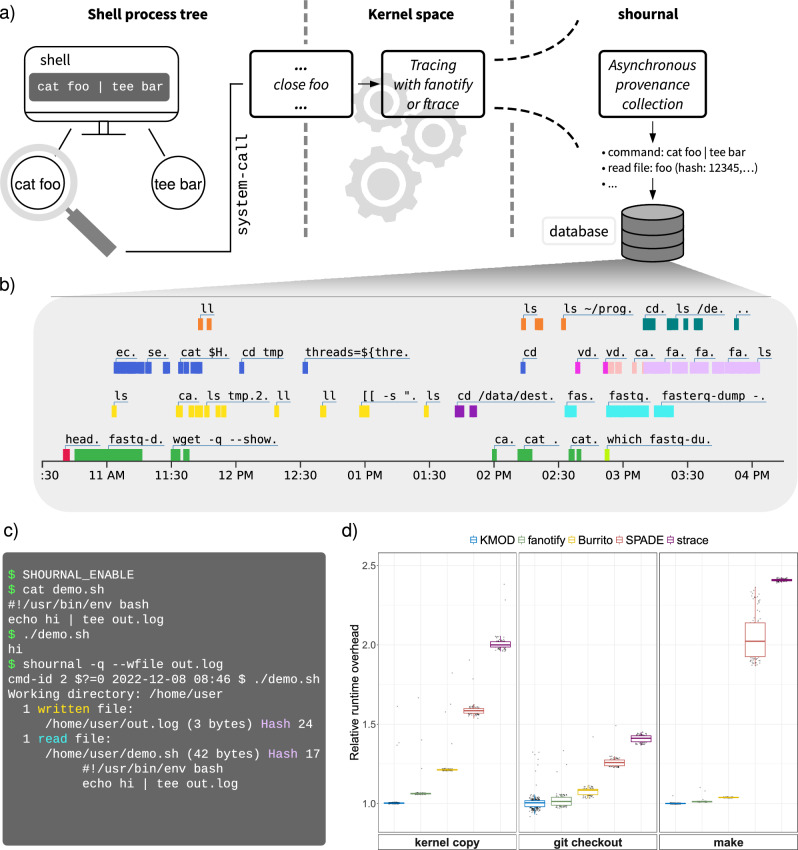
Shournal records shell commands and used files. (**a**) Schematic illustration of a shell session observed by *shournal*. The whole process tree of the command cat foo $\vert$ tee bar is monitored for file-close system calls. When foo is closed, the event is traced in kernel space within process context, while the asynchronous provenance collection runs in another thread. Finally metadata and checksums of the used files are stored alongside the corresponding shell command within *shournal*’s database. (**b**) Visualization of the shell command history. *shournal* can export the command history into an interactive Html-plot. Commands, which were executed within a given shell session, are marked with the same color. Parallel shell sessions are vertically stacked. (**c**) An example shell session observed by *shournal*. The executed script demo.sh creates a file out.log. Next, *shournal*’s database is queried for commands which created or modified out.log using its –wfile option. *shournal* reports the command, file checksums and the archived script. (**d**) The impact of tracing on runtime performance in various scenarios, given as the ratio of a monitored process over an unmonitored process. Boxes for both *shournal*-backends, kernel module (KMOD) and fanotify, are displayed. For comparison, our measured tracing overhead of *Burrito*, SPADE and *strace* is shown as well.

: first, tracing is implemented using *ftrace* and *tracepoints* from within *shournal*’s own kernel module (KMOD), or an alternative backend instrumenting the kernel-builtin *fanotify* filesystem API, both orders of magnitude faster than *ptrace*^15^. Second, tracing of file actions is limited to the comparatively seldom close operation and lets the traced process return quickly by delegating further provenance collection to another thread (Fig. 1a). Third, by default, *shournal* primarily captures file metadata such as path, size, modification-time, and (partial) checksums, so the amount of data read from and stored to disk is small.

Besides logging metadata, *shournal* can be configured to archive whole scripts or configuration files for dedicated directories or file extensions. To prevent, e.g., a backup script from flooding *shournal*’s database, the maximum filesize and the number of archived scripts, as well as the maximum number of file events per command, is configurable.

To make use of the collected data, *shournal* provides flexible and fast (typically less than a second) queries on the command line. For instance, for a given file, the exact shell commands that created it or used it can be retrieved (Fig. 1c). Archived scripts or configuration files are restorable, enabling a re-execution of commands, even if the original files have long been modified or deleted. File checksums of previous invocations allow for a comparison to the current state, so, in case a command execution suddenly yields different results, it can quickly be determined which input files or scripts changed. Other options include queries for files modified during a given period, the command history of a project directory, or commands executed during a specific shell session. Such queries may be useful for virtually all scientists working on the shell, especially when applications with various input options are used. An example from the field of computational chemistry is *OpenChem*^16^, a deep learning framework based on PyTorch^17^. Parameters of the deep learning models, like the number of epochs or the used optimizer, are defined in configuration files whose values are partly overridable via the command line. *shournal* can track and restore both in conjunction, simplifying the re-execution of the experiment at a later time. Similar applies, e.g., to the *Ensembl Variant Effect Predictor*^18^, a tool used in bioinformatics.

In addition to the console output, *shournal* generates an interactive graphical map of commands for user-specified files, directories, or dates (Fig. 1b). The map displays each shell session in an individual row, allowing one to identify specific chains of subsequently executed commands better. Clicking on a command displayed in the interactive map gives Supplementary Information on the exact execution time, archived scripts, or checksums.

The collected data can be shared with other programs using the JSON format. Due to the low-level nature of the data, it can be used as a basis for higher-level systems such as workflow managers. For instance, rule templates for the *Snakemake workflow engine*^3^ can be generated from an observed series of shell commands using the software at https://github.com/snakemake/shournal-to-snakemake. The input- and output section of a rule is generated from the captured file events.

Thereby, *shournal* allows one to comprehensively record, search and visualize the work carried out on the shell with configurable low runtime- and storage overhead. It alleviates the reproducibility of scientific analyses by permitting the reconstruction, summary, and resumption of projects quickly. With the *shournal-to-snakemake* converter, we demonstrate that the collected data may be used as a basis for the creation of workflows. *shournal* does not require a self-compiled kernel and is easy to install (Debian packages and other binaries are provided along with the source code at https://github.com/Hoffmann-Lab/shournal). Observation of all the shell sessions of a user can be started by adding two lines of code to the shell’s rc (e.g. .bashrc).

## Discussion

To our knowledge, *shournal* is currently the only readily available tool to track all the user’s shell commands and their used files with low overhead, irrespective of the underlying filesystem. Two backends, using either fanotify or ftrace, are provided to cater to different administrative needs. By storing the data at the granularity of individual commands and their used files, *shournal* supports the creation of pipelines and toolchains without requiring the user to make any specific provisions. This may be relevant in all situations where the assembly of a pipeline was not initially intended. Also, it helps all users not yet familiar with the use of sophisticated workflow engines to keep track of their work and still be able to create a proper workflow later on. Our *shournal-to-snakemake* converter, generating basic *Snakemake* rule templates from the user records, demonstrates how *shournal* may help to facilitate some of this work. Additionally, if a version control system is not in use, *shournal* may enhance the reproducibility of workflow executions by restoring scripts or other input files from previous runs. By closing the critical gap between ad-hoc work on the shell, its manual documentation, and the creation of workflows, also those researchers already using workflow solutions and tools such as *CDE*, *CARE*, or *ReproZip* can benefit. In this way, *shournal* supports good scientific practice.

In its current implementation (version 3.1), *shournal* still lacks some desirable features. Specifically, when calculations are distributed over multiple machines, the file-event history may have some gaps. While provenance is recorded on all devices where *shournal* is installed, reconstructing the history is more challenging in this case. Files opened read-writable are currently handled as write-only files. Although, in practice, many workflows separate input- and output files, there are certainly exceptions. Both features shall be appropriately addressed in upcoming versions of *shournal*. Additional detailed requirements and limitations are discussed in the Supplementary Material.

As of *shournal* version 3.1, only Linux is supported natively. However, users of Microsoft Windows can use the *fanotify edition* via the Windows Subsystem for Linux (WSL). Future versions shall also add native support for other operating systems, e.g., Microsoft Windows or MacOS.

Finally, while *shournal* supports users by tracking how they did their work, it does not relieve them from carefully documenting the rationale for a particular solution or their reasoning about it.

## Methods

### Tracing

*Shournal* implements its design goal to associate shell commands and their used files through kernel space tracing. Specifically, *shournal* installs a global hook within the Linux kernel, which runs whenever any process on the system closes the last instance of a file descriptor. Within that hook, it is determined if the executing process is part of an observed shell command, whereupon a raw reference on the file path (a kernel object, not a string) is taken and buffered. This allows the monitored process to resume quickly while further processing steps run asynchronously in another thread. *Shournal* provides two backends to trace file close events of specific processes: its own kernel module *shournalk* and another, *fanotify*-based backend.

*Shournalk* is based on tracepoints and the *ftrace* framework, allowing to run custom code at specific execution paths without kernel recompilation^15^. Only three events are traced. In addition to tracing the closing of files, the *exit* of processes and *fork* events are kept track of. Tracing of the latter allows us to maintain a list of shell processes and their descendants. A process can initially be marked for observation using *shournalk*’s sysfs-interface^19^.

The *fanotify*-based backend employs the kernel-native *fanotify* filesystem API to register for file close-events and does not involve a kernel modification. However, while *fanotify* allows event subscriptions for whole mount points^20^, it provides no direct way to monitor only file events of specific processes. To remedy this shortcoming, for each shell command, *shournal* creates a unique unshared mount namespace^21^ and ensures that file operations of the shell and its child processes refer to it.

The *fanotify*-based backend provides a shared library, hooked into the shell’s process, and a setuid (*suid*) program^22^ performing the privileged actions of unsharing and joining mount namespaces as well as marking mount points with *fanotify*. Specifically, the shared library masks the library calls *open* and *execve*. The former ensures that files within the parent shell and its subshells are opened relative to the new mount namespace, while the latter redirects to *shournal*’s *suid* binary, which performs the privileged *setns* syscall to join the new mount namespace, executing the original program afterward. The mount-namespace is, by default, inherited during *fork*, notable exceptions being, e.g., container solutions like *Docker*. As a result, applications unsharing the mount namespace themselves cannot be traced from an outer layer. However, *shournal* provides a dedicated *Docker* edition which does allow a shell running within *Docker* to be observed.

While *shournalk* is faster (see section “Performance”), has fewer limitations, and alters the user-space environment to a lesser extent, the *fanotify* backend may be of particular interest to institutions where the installation of foreign kernel modules is discouraged.

#### Provenance collection

An asynchronous provenance collection thread consumes the file close events, which were buffered for observed shell processes and their children. It filters files according to the user’s preferences, e.g., by archiving only scripts ending with *.sh* or ignoring events from the system’s temporary directory. To ensure the identity of a given file, beyond metadata like name, size, and modification time, a checksum of the file content is calculated using *xxHash* https://github.com/Cyan4973/xxHash. As hashing large files in their entirety could introduce a considerable slowdown, only *N* chunks of length *b* bytes of a file are digested at regular offsets calculated from the file size *s*. Starting from the beginning of a file, the seek-step *p* is $p = \lfloor \frac{s}{N} \rfloor$ bytes. Small files, where $p \le b$, are hashed completely. Three chunks of 256 file bytes are digested by default, keeping the overhead low. On the downside, files of equal size with the same content in the hashed regions but different content in others are falsely reported as equal. While we did not encounter such an error in practice, *shournal*’s hash settings remain configurable to digest more file parts, if appropriate. Obtaining the correct checksum of a file’s “final” version is guaranteed to be free from race conditions as long as all writers are part of the observed process tree: at some point, the last writer closes the file, after which the final hashing is performed. Size, checksum, and optionally modification time allow for later file provenance queries with high accuracy independent of the filename. Thus, *shournal* abstains from tracing rename operations.

In the first instance, to be fast and lightweight, metadata and archived scripts are stored in a binary, partially compressed file and later, without time pressure, finally stored in an SQLite database by a low-priority background daemon. The maximum number of archived scripts and file events per command is configurable, so, by default, a backup script cannot flood *shournal*’s database. To further reduce disk usage, files are archived in a deduplicated manner.

#### Performance

To analyze *shournal*’s performance, we designed a benchmark reflecting scenarios with intensive file access. During *kernel copy*, for instance, more than 120,000 files were read and written within a few seconds. In addition, we benchmarked a *git checkout* of the Linux kernel source code and the compilation of *elfutils* with *make*. For each command, the time of a “cold cache” run was measured at least 100 times, while between each run, the file cache of the Operating system (OS), as well as the disk cache, was cleared.

We find that the median runtime overhead is less than 0.5% for the kernel module backend and less than 6.3% for the *fanotify* backend in all examined cases (Fig. 1d). These results demonstrate the applicability of our tool.

For comparison with *Burrito*, we benchmarked its *SystemTap*-script^11^. The runtime overhead of partially exceeding 20% already occurs due to plain tracing and event logging. No further processing or file-versioning using the NILFS filesystem was involved, so we consider this the lower bound of the performance penalty one has to expect when using this tool.

For comparison with SPADE, we installed and configured its *auditd* reporter ( add reporter Audit). The runtime overhead of partially exceeding 100% already occurs due to plain tracing and event processing (without disk logging), so we conclude that the runtime overhead might be even higher in practice. Note that we did not measure SPADE’s *camflow* backend due to our initial requirement of not depending on a self-compiled kernel.

Finally, as a representative for *ptrace*-based tools, we measured runtimes under the tool *strace*^23^ and found large overheads of partially exceeding 140%.

The storage overhead of *shournal*’s database critically depends on the user configuration. Using default settings, the cp-benchmark yields a disk usage of 174 bytes per file event. Thus, 1 GiB of disk space is sufficient for recording approx. 6 million events. For a regular shell user, we estimate a storage requirement of less than 100 MiB per month. Furthermore, *shournal* provides a rich command line interface to delete unneeded entries, for example, by age (e.g., older than 2 years) or by project directory.

Hardware: All tests were run on an Intel(R) Xeon(R) CPU E-2146G CPU with six cores, 31 GiB RAM, and a 1 TB PC601 NVMe SK hynix SSD.

Software and settings: OS was openSUSE Leap 15.1 with non-default kernel 4.12.14-lp151.28.91. The old kernel version was used for comparison with *Burrito*, whose *systemtap*-script from 2012 does not run on more recent kernel versions. The benchmark used a dedicated disk to reduce the impact of OS activity on the results. For the same reason, multi-queue disk access^24^ was enabled. Native command queuing (NCQ) was disabled due to potentially large, don-deterministic delays and vendor-specific implementation^25^. To make benchmark executions comparable and stable, they were executed at the highest non-turbo CPU-frequency with Hyper-threading disabled. As modern processors often control the frequency themselves, rather than following user requests, cores were kept near their maximum frequency using the idle=poll kernel parameter. The CPU scaling driver intel_pstate was disabled in favor of the older acpi-cpufreq driver to reduce noise, as reported by^26^. Each benchmark iteration started with a “cold” cache: disk cache was cleared by reading 1.5 GB of unrelated data from the benchmark drive, and the OS page cache^27^ was evicted by re-mounting the respective partitions after each run. The full Grub command line was: idle=poll intel_pstate=disable intel_idle.max_cstate=0 processor.max_cstate=1 scsi_mod.use_blk_mq=1 libata.force=noncq,noncqtrim systemd.unified_cgroup_hierarchy=0.

The tracing tools were used in the following versions: *shournal* (v2.9), *Burrito* (commit 6630cb2), SPADE (commit 3437fcd), strace (v4.20). For the *kernel copy* the Linux source code v4.19.132 was copied with the *cp* command. During *git checkout*, the Linux git tree, hard-reset to v4.19, was checked out to v3.10. Finally, *make* was executed on elfutils v0.176 using configure  && make -j$(nproc).

Due to the relatively small overhead of the kernel module backend, 300 repetitions were performed for each command. All other backends (including *fanotify*) were configured for 100 repetitions. In all cases, runtimes were recorded after seven “warmups”. To compensate for potential long-lasting cache effects, traced and untraced runs were carried out alternating, while, for each execution pair, it was randomly sampled which to run first.

*Shournal* was configured to impose no limit on the number of logged read and written files, to store at most ten read scripts ending with the suffix *.sh*, and not exceeding a size of 0.5 MiB and to calculate partial file checksums with the default of at most 3 ${{\times }}$ 256 bytes. As described previously, events are logged to an intermediate binary file during tracing, while the final storing to the sqlite database happens later. Therefore, that final data transfer was not part of the runtime measurements. We consider this benchmark design reasonable since, after the time-critical recording phase, the collected provenance can be held indefinitely long and is permanently stored using a low-priority background thread.

Event logging was performed on the same disk where the respective files of the benchmark resided, constituting a worst-case.

### Supplementary Information

Supplementary Information.

## Data Availability

shournal’s code and binaries are freely available at https://github.com/Hoffmann-Lab/shournal under the GNU General Public License v3.0 or later and other open-source licenses. Archived for this publication, version v3.1 can also be obtained from https://doi.org/10.5281/zenodo.10473782.
