# Supplementary material for: Bashing irreproducibility with shournal
Source: Sci Rep. 2024 Feb 28;14:4872. doi: 10.1038/s41598-024-53811-9 (PMC10901821; doi:10.1038/s41598-024-53811-9)
Supplement: Supplementary file 1 — Supplementary Information. [file 41598_2024_53811_MOESM1_ESM.zip › supplement.pdf]

Bashing irreproducibility with *shournal* - Supplementary material

Tycho Kirchner[1], Konstantin Riege[1], and Steve Hoffmann[1,*]

[1]Computational Biology Group, Leibniz Institute on Aging — Fritz Lipmann Institute (FLI), Jena, 07745, Germany
[*]steve.hoffmann@leibniz-fli.de

ABSTRACT

Arguably, the most important tool for many computational scientists is the Linux shell. Processing steps carried out there are critical for a large number of analyses. While the manual documentation of the work is time-consuming and error-prone, existing tools do not integrate well into the shell or suffer from a large overhead. Here, we present *shournal*, which integrates tightly into the shell and automatically records all shell commands along with their associated file events. Thus, for all files, it can later be told how they were generated and processed. Additionally, it allows the creation of detailed reports for whole project folders. *shournal* retrieves its data directly from the Linux kernel and allows the monitoring of whole process trees with low overhead.

1 Additional Limitations

Tracking is currently not possible when the observed process A instructs the unobserved process B via interprocess communication (IPC)[1] to use a file. For shell workflows this is rather uncommon, however, if it proves to be an issue, future versions of *shournal* shall allow to execute B in the provenance-context of A.

In cases of extremely high file event frequencies of a single process tree *shournal* may drop some events to avoid slowing down the process, loosing the provenance of individual files. Apart from artificial benchmarks, we observed this rarely for the *fanotify* backend and never for the kernel module backend.

Memory mapped[2] files are currently only tracked correctly if the underlying file descriptor is closed after (and not before) usage. While this is good practice, it is, technically, not required by Linux. Thus, events evoked by programs that do not properly close the descriptor may be missed.

In case of the *fanotify*-backend the observation of a process tree ends when the last instance of a dedicated file descriptor, inherited by child processes[3], is closed (which happens automatically on exit). If processes do not wait on their children **and** explicitly close this file descriptor, subsequent file-events are lost. Further, as process trees are isolated against each other using unshared mount namespaces, file provenance for traced programs which unshare the mount namespace themselves is lost. The kernel module backend does not suffer from these limitations and can be used instead, if necessary.

When a dedicated file descriptor is opened during interactive sessions (e.g. via `exec 3> some-file`) and used within separately entered commands, only the last command using it before closing is associated with that file event. Background is that *shournal* only tracks close events when the last instance of a given file descriptor is closed. The same applies, e.g., when the descriptor is sent via *unix domain sockets*[4] to an unobserved process and the final closing happens there. In practice, however, this should not be an issue.

2 Applicability of shournal

To get an intuitive understanding of the applicability of our tool, we provide a simple usage example.

Scientist Sarah creates a new pipeline for her current project. As this work involves a lot of trials and a careful examination of each intermediate result, all of the steps are first carried out on the interactive shell. In the middle of the process, her collaborator asks her for another short but urgent analysis. Sarah does this and returns to the initial project. Unfortunately, she has no idea where exactly she left off. If she is lucky, the last steps are still within the shell's history. This reconstruction, however, involves searching through unrelated commands executed in between. Fortunately, Sarah organizes her work in project directories and can quickly ask *shournal* what she did last in that particular working directory (cwd). By typing `shournal -q -cwd $PWD -history 50`, Sarah can recall the last 50 entered commands, including a brief summary of the read and written files. Sarah then quickly resumes and finishes the analysis. Finally, she wants to automate the steps by writing a shell script.

Initially, she aligned RNA-sequencing reads with a certain accuracy threshold, which resulted in a file `rna.bam`. But which threshold was it exactly? The shell's history shows she tried a good dozen different ones. Since *shournal* was previously

configured to capture the provenance of all files written to that project directory, she is able to answer this question very quickly. Executing `shournal -q -wf rna.bam` yields exactly the shell command that led to the creation of this file and unveils the threshold 95. Of note, the `-wf`-(written-file-)argument ensures that this command works irrespective of the filename `rna.bam`, as the file is found in *shournal*'s database by its metadata (size, modification time) and hash. Next, Sarah executes the whole script, and all goes well. The next day she remembers documenting her work and creating an archive of the experiment using, e.g., ReproZip[5]. Unfortunately, the result is suddenly different from the previous run. While this was the beginning of a frustrating process in the past, finding the reasons for this difference is more straightforward with *shournal*. By calling a *diff* on the read files of the two executed commands, it becomes clear that a co-worker modified a common script which is part of Sarah's pipeline. Since Sarah configured *shournal* to store script files (files ending with .sh) within its database, reproducing her previous result takes only minutes. Even in cases where the whole script file itself was not archived, the different metadata (size, modification time) and the hash of a changed read or written file is a strong hint where action is needed. Finally, Sarah does not only work on the shell but also with the workflow manager *Snakemake*[6] for recurring tasks. Using the converter https://github.com/snakemake/shournal-to-snakemake, the process of transforming the existing pipeline into a *Snakefile* is simplified.

References

1. Kerrisk, M. In *The Linux Programming Interface*, 877–878 (No Starch Press, 2010).

2. Kerrisk, M. In *The Linux Programming Interface*, 1017–1019 (No Starch Press, 2010).

3. Kerrisk, M. In *The Linux Programming Interface*, 29–30 (No Starch Press, 2010).

4. Kerrisk, M. In *The Linux Programming Interface*, 1165–1166 (No Starch Press, 2010).

5. Chirigati, F., Rampin, R., Shasha, D. & Freire, J. ReproZip: Computational Reproducibility With Ease. In *Proceedings of the 2016 International Conference on Management of Data*, SIGMOD '16, 2085–2088, DOI: 10.1145/2882903.2899401 (Association for Computing Machinery, New York, NY, USA, 2016).

6. Köster, J. & Rahmann, S. Snakemake—a scalable bioinformatics workflow engine. *Bioinformatics* **28**, 2520–2522, DOI: 10.1093/bioinformatics/bts480 (2012).
