# Supplementary material for: Bashing irreproducibility with shournal
Source: Sci Rep. 2024 Feb 28;14:4872. doi: 10.1038/s41598-024-53811-9 (PMC10901821; doi:10.1038/s41598-024-53811-9)
Supplement: Supplementary file 1 — Supplementary Information. [file figure1_v12.pdf › figure1_v12.pdf]

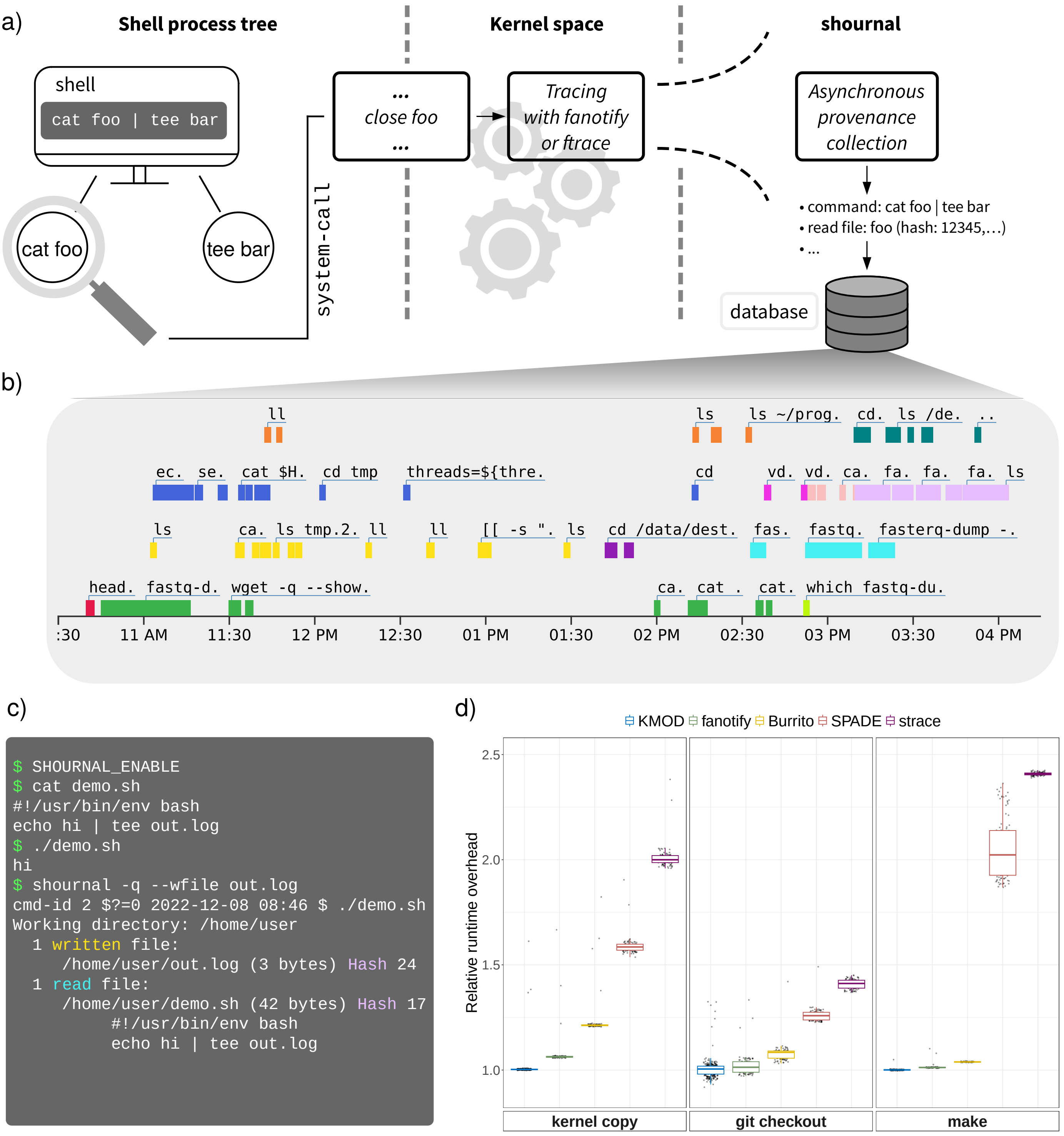

a)
Shell process tree
Kernel space
shournal
shell
cat foo | tee bar
cat foo
tee bar
system-call
...
close foo
...
Tracing
with fanotify
or ftrace
Asynchronous
provenance
collection
• command: cat foo | tee bar
• read file: foo (hash: 12345,...)
• ...
database
b)
ll
ls
ls ~/prog.
cd.
ls /de.
..
ec.
se.
cat $H.
cd tmp
threads=${thre.
cd
vd.
vd.
ca.
fa.
fa.
fa.
ls
ls
ca.
ls tmp.2.
ll
ll
[[-s ".
ls
cd /data/dest.
fas.
fastq.
fasterq-dump -.
head.
fastq-d.
wget -q --show.
ca.
cat .
cat.
which fastq-du.
:30
11 AM
11:30
12 PM
12:30
01 PM
01:30
02 PM
02:30
03 PM
03:30
04 PM
c)
$ SHOURNAL_ENABLE
$ cat demo.sh
#!/usr/bin/env bash
echo hi | tee out.log
$ ./demo.sh
hi
$ shournal -q --wfile out.log
cmd-id 2 $?=0 2022-12-08 08:46 $ ./demo.sh
Working directory: /home/user
1 written file:
/home/user/out.log (3 bytes) Hash 24
1 read file:
/home/user/demo.sh (42 bytes) Hash 17
#!/usr/bin/env bash
echo hi | tee out.log
d)
KMOD
fanotify
Burrito
SPADE
strace
Relative runtime overhead
2.5
2.0
1.5
1.0
kernel copy
git checkout
make
